# Revisiting the Term “Compassionate Use” and Leadership of the World Health Organization in Resolving Confusion in the Age of COVID-19 and Beyond

**DOI:** 10.31662/jmaj.2022-0053

**Published:** 2022-08-01

**Authors:** Akio Teraoka, Shunsuke Ono, Ryuichi Ida, Kiichiro Tsutani

**Affiliations:** 1 Department of Pharmaceutical Regulatory Science, Graduate school of Pharmaceutical Sciences, The University of Tokyo, Tokyo, Japan; 2 The Japan Association of National Universities (JANU), Tokyo, Japan; 3 The Institute of Seizon and Life Sciences (ISLS), Tokyo, Japan

**Keywords:** compassionate use, expanded access, unapproved drugs, emergency use, infectious disease outbreak, World Health Organization, terminology, regulation

## Abstract

We discuss the term “compassionate use” (CU) as an example of terminology having a huge impact on drug regulation. CU is used in many confusing situations, and its meaning varies significantly.

We ethically affirm the necessity of CU. We insist that CU should be properly placed in exceptional status. The regulation of CUs is much more lenient than that of clinical trials because of the difference in the purpose. Whether consciously or unconsciously, abuse results in confusion and is never acceptable.

The World Health Organization (WHO) proposed not to use the previous term CU but to replace it with another one. WHO also proposed the term MEURI (monitored emergency use of unregistered and experimental interventions). However, this was extremely incomplete, and WHO used the term CU subsequently. The main purpose of the proposal needs to be thoroughly implemented.

In the context of the COVID-19 pandemic and beyond, expectations regarding WHO’s role and leadership in global health issues are rising. We hope that WHO will play a major role in promoting research ethics preparedness while discontinuing the use of confusing terms such as CU and will develop alternative terms and their content.

We discuss the evaluation of MEURI, the Japanese version of CU, and appropriate and inappropriate terminology related to the therapeutic use of unapproved drugs. We also discuss the expected appearance of CU including its name. It is appropriate to target group/cohort patients and unapproved drugs in the late stage of development. It is also important to solve the problem of incentives for CUs of pharmaceutical companies that are rushing to obtain marketing approval. The UK’s Early Access to Medicine Scheme has provided many suggestions.

We believe that our opinion can contribute to WHO’s efforts to resolve the confusion and promote research ethics preparedness in health emergencies.

The confusion over terminology in healthcare has a huge impact on the regulation of drugs and thus requires drastic improvements. A typical example would be the term “compassionate use” (CU). The US Food and Drug Administration (FDA) officially used the term “expanded access” (EA) instead of CU in 1987. CU is used in many confusing situations, and its meaning varies significantly ^(1), (2)^.

The World Health Organization (WHO) proposed the term “monitored emergency use of unregistered and experimental interventions” (MEURI) in 2014 as an alternative to CU in the context of the debate over ethical issues related to the therapeutic use of unapproved drugs during the Ebola pandemic ^(3)^. However, the related guidelines were extremely incomplete. WHO used the term CU subsequently. It is significant that in 2014, WHO proposed not to use the previous term CU but to replace it with another one. The main purpose of the proposal needs to be thoroughly implemented.

WHO’s term MEURI is intended for emergency use of unapproved drugs when they are not ready to begin clinical trials (CTs). As the term implies, monitoring is important and intervention results are recorded. WHO also stated that “such emergency use should not preclude or delay the initiation of more conclusive investigations of interventions in properly designed clinical studies. The latter, if appropriately designed and executed, may yield generalizable conclusions that result in greater societal benefit.” WHO has been requested to create a more detailed guideline on the use of MEURI, but it has not yet been published. After WHO proposed the use of MEURI, the unprecedented COVID-19 pandemic occurred at the end of 2019, enhancing the need for effective and safe drugs. The acceleration speed of CTs and RCTs is remarkable. WHO’s SOLIDARITY trial and the United Kingdom (UK)’s RECOVERY trial are typical examples of RCTs. The use of MEURI has been suggested to be limited due to these changes in the situation, and it is necessary for CUs to become what they should be instead of MEURI.

The FDA website refers to EA as “a potential pathway for a patient with an immediately life-threatening condition or serious disease or condition to gain access to an investigational medical product for treatment outside of CTs when no compatible or satisfactory alternative therapy options are available,” which is different from CT. The European Medicine Agency officially uses the term CU. Its website also states the same intended meaning as the FDA, although, unlike the FDA, the program targets only a group of patients, and CU is a separate entity from CT. However, many member states of the European Union do not distinguish CU from CT. Furthermore, CU refers to the therapeutic use of unapproved drugs even when CT is not performed ^(1), (2)^.

The fact that the use of unapproved drugs is mentioned in the word “compassion” suggests that access through CTs is the main principle and CUs are examples of exceptional and special cases. We ethically affirm the necessity of CU. To resolve the confusion, we insist that CU should be properly placed in this status. The purpose of CTs is to accumulate the data necessary for drug approval, whereas CU gives priority to using unapproved drugs for treatment. This difference in purpose makes the regulation of CUs much more lenient than that of CTs. Abuse of this looseness, whether consciously or unconsciously, is a major source of great confusion and is never acceptable.

In Japan, the introduction of CU programs, which exist in Europe and the US, has been delayed, and the safety of the use of unapproved drugs personally imported from abroad has become a problem. In January 2014, the Japanese government founded a Japanese version of the CU program ^(4)^ that enables access to promising drugs in the late stage of development for a group of patients. It should be noted that this program was created within the framework of a “Chiken,” which is a CT. “Chiken” refers to CTs conducted by pharmaceutical companies in accordance with Good Clinical Practice to obtain marketing approval and bundled reimbursement. The program enables the provision of unapproved drugs in CTs in Japan to patients who do not meet the criteria for participation in CTs from a humanitarian point of view. Thus, in Japan, CU is not a separate entity from CT.

This program seems far from being established. This can be seen in the fact that the implementation status published on the website ^(4)^ is only a very small part of the number of CTs published at the same time. This suggests that conducting CUs as CTs causes an excessive burden on the practitioner, which in turn impedes patient access. Although the creation of the program within the framework of CTs helped to start the program earlier, we believe that it was unreasonable to combine CU and CT, which have different objectives. In the future, we hope that the program will be developed as a system specializing in exceptional and special access.

Kimberly et al. ^(1)^ proposed that the use of CU and EA―as terminology for the therapeutic use of unapproved drugs―has been abandoned despite its historical usage and inherent appeal. They also proposed that as an umbrella term, preapproval access should be used as a neutral term and individual/named patient access and group/cohort patients’ access should be used as a regulatory route for preapproval access. We support the terminology used by Kimberly et al. Hope ^(5)^ responded to “the slippery slope argument” by drawing a line or placing a barrier at some stage along the slope. “The precise drawing of the line is arbitrary; but it is not arbitrary that a line is drawn. To ensure clear policy (and clear laws), it is often sensible to draw precise lines even though the underlying concepts and moral values change more gradually.” The distinction between individual/named patient access and group/cohort patients’ access can be considered.

However, in our opinion, as a regulatory route for preapproval access, only group/cohort patients’ access is appropriate and individual/named patient access must incline toward a case-by-case basis. We summarize appropriate and inappropriate regulatory terminology to be used for access to unapproved drugs in emergencies, such as infectious disease outbreaks and other health emergencies, in Table 1.

**Table 1.**
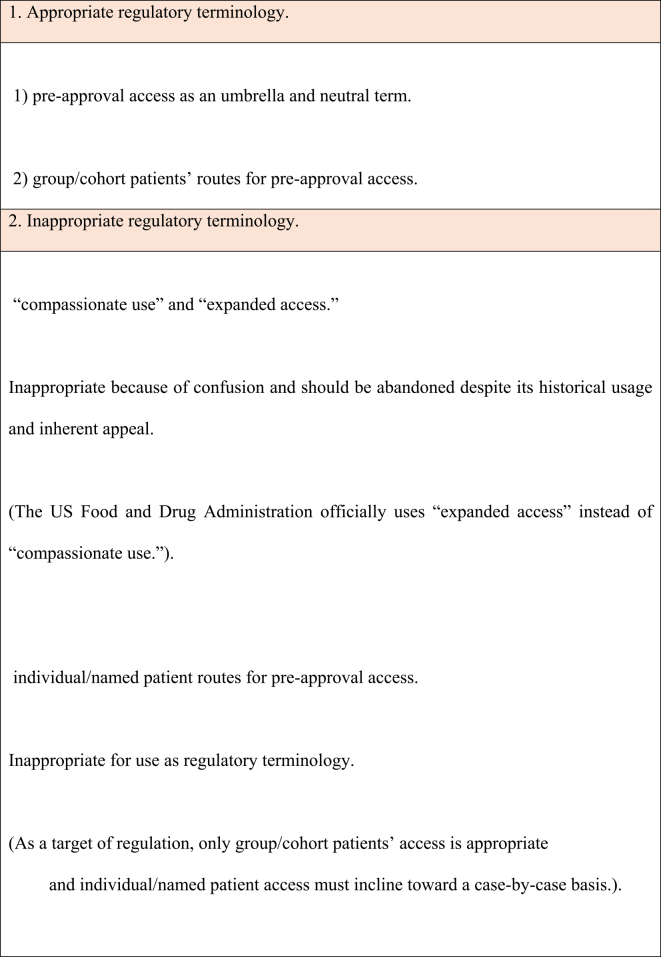
Classification of Appropriate and Inappropriate Regulatory Terminology.

Another important point about the CU program is its concrete form. In particular, two important issues are selecting appropriate drugs for the program and the incentive challenges for pharmaceutical companies that are rushing to obtain marketing approval based on the accumulation of CT and RCT data. The UK’s Early Access to Medicine Scheme (EAMS) has provided many suggestions for the expected appearance of CU. This scheme was established by the UK government in 2014 under an agreement with pharmaceutical companies to provide early access to breakthrough drugs. EAMS can be described as a scheme that incorporates CU into the development process of promising drugs in the later stage of development. The drugs will be provided free of charge by the companies, but they will be linked with the company’s own promising innovative medicine application at EAMS, and the promising drug application will be facilitated for early approval and early insurance reimbursement. It is a scheme that aims to improve the quality of target drugs, to avoid hindering or delaying the implementation of RCTs, and to solve the problem of incentives for companies, which is often a concern.

The name of the desired CU is also important. It could be called a special early access scheme (SEAS). This name was coined by us for the first time, but we think that it is appropriate because it is not an inorganic name and we can imagine exceptional and special access to unapproved drugs.

In the context of the COVID-19 pandemic and beyond, expectations regarding WHO’s role and leadership in global health issues are rising. In March 2018, the WHO Global Health Ethics Team and the African coaLition for Epidemic Research, Response, and Training (ALERRT) launched the ALERRT-WHO workshop to promote research ethics preparedness in infectious disease outbreaks and other health emergencies. The workshop expects WHO to take a leadership role in its implementation. They also emphasize the importance of global clarification of the terminology used in ethics preparedness ^(6)^. The COVID-19 pandemic and beyond is an excellent opportunity for radical improvement. During this unprecedented global infectious disease outbreak crisis, the expectations for WHO’s role are rising, and we look forward to WHO’s leadership. We summarize the expectations for WHO’s leadership in resolving the confusion in the age of the COVID-19 pandemic and beyond in Table 2.

**Table 2.**
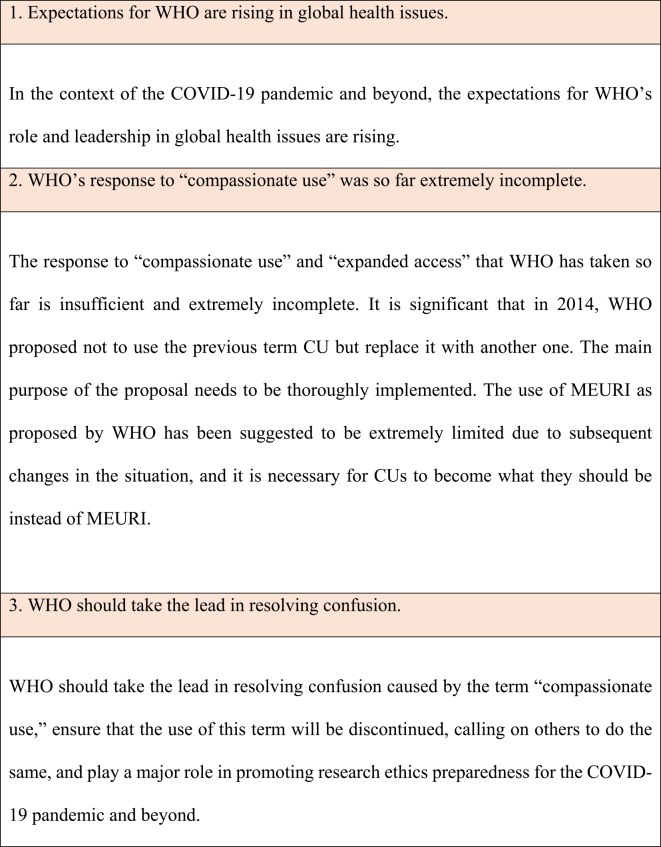
Expectations for the World Health Organization (WHO)’s Leadership in Resolving Confusion.

To the best of our knowledge, this is the first opinion that affirms the proper position of the so-called CU and shows how it should be. It is also the first request for WHO to take leadership in resolving the confusion caused by CU and EA.

We believe that our opinion can contribute to WHO’s efforts to resolve this confusion.

## Article Information

### Conflicts of Interest

None

### Author Contributions

AT, SO, RI, and KT are equal contributors to the study. AT wrote the manuscript as the first author. SO, RI, and KT reviewed the manuscript. All authors read and approved the final manuscript.

### Approval by Institutional Review Board (IRB)

Not applicable
